# The Relevance of TLR8 in Viral Infections

**DOI:** 10.3390/pathogens11020134

**Published:** 2022-01-22

**Authors:** Iván Martínez-Espinoza, Antonieta Guerrero-Plata

**Affiliations:** Department of Pathobiological Sciences, Louisiana State University, Baton Rouge, LA 70803, USA; imart27@lsu.edu

**Keywords:** TLRs, TLR8, TLR7, virus, viral infections, TLR agonists

## Abstract

Toll-like receptors (TLRs) are the largest pattern recognition receptors responsible for activating the innate and adaptive immune response against viruses through the release of inflammatory cytokines and antiviral mediators. Viruses are recognized by several TLRs, including TLR8, which is known to bind ssRNA structures. However, the similarities between TLR8 and TLR7 have obscured the distinctive characteristics of TLR8 activation and its importance in the immune system. Here we discuss the activation and regulation of TLR8 by viruses and its importance in therapeutical options such as vaccine adjuvants and antiviral stimulators.

## 1. Introduction

A critical component of innate immunity in the defense against invading pathogens are the pattern recognition receptors (PRRs) [1], which are germ-line encoded proteins with a refined ability to distinguish pathogen-associated molecular patterns (PAMPs) present in several types of microorganisms.

Toll-like receptors (TLRs) are the largest class of PRRs that initiate and regulate host immunity by activating the innate and subsequent adaptive immune responses [2]. Currently, 10 human TLRs have been identified at different cell locations and are widely distributed among different cell types. TLRs are type I membrane glycoproteins that can be divided into two groups based on their location and capability of recognition, and they can also be homo- or heterodimers [3]. TLR1, TLR2, TLR4, TLR5, TLR6, and TLR10 are located on the cell membrane, whereas TLR3, TLR7, TLR8, and TLR9 are found in the endosomes [3,4,5].

There are multiple PAMPs identified as the specific ligands for each human TLR. Therefore, TLR1, TLR2, and TLR6 recognize lipoproteins on microbial cell walls; TLR5 recognizes flagellin, while TLR4 recognizes lipopolysaccharides (LPS) and other microbial structures. In addition, TLR10 can recognize bacterial and viral proteins [3,6,7]. Finally, endosomal TLRs bind nucleic acids, where TLR3 recognizes dsRNA, TLR9 recognizes bacterial and viral DNA, and TLR7 and TLR8 are reported to bind ssRNA. However, more recent evidence highlights critical differences between TLR7 and TLR8 by indicating that their activation triggers distinct signaling pathways and induces differential cytokine profiles [8]. Nonetheless, the study of TLR8 has not been as robust as that of TLR7.

## 2. Activation and Regulation of TLR8

**Figure 1:**
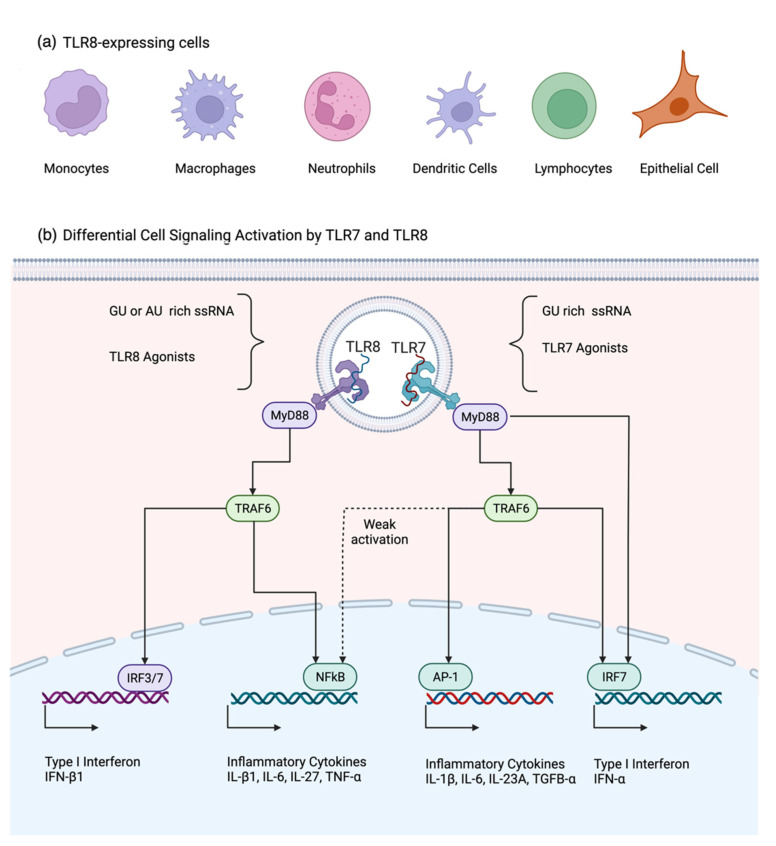
(**a**) Cells that are reported to express TLR8 under basal conditions; (**b**) TLR7 and TLR8 signaling pathways have the common adaptor protein MyD88. IFN-α expression after TLR7 can be achieved by forming the complex MyD88-IRF7 or by the activation of IRF7 via TRAF6. TRAF6 also activates AP-1 and weakly NF-kB, promoting inflammatory cytokine expression. TLR8 induces a more potent inflammatory cytokine response than TLR7 via the activation of TRAF6-NF-kB pathway, while the induction of IFN-b by TLR8 is achieved through the activation of the IRF3/7 pathway. Created with BioRender.com. (Accessed on 17 December 2021.)

Human TLR8 is expressed in monocytes, macrophages, neutrophils, myeloid dendritic cells [3,6,8,9,10], and regulatory T (Treg) cells [11] (Figure 1a). TLR8 is a type I transmembrane receptor characterized by three structural components: an extracellular domain-containing leucine-rich repeats (LRRs), a transmembrane domain, and a cytoplasmic Toll-interleukin 1 receptor (TIR) domain [12]. Its extracellular domain is integrated by approximately 800 amino acids and 26 LRR modules and allows TLR8 to recognize ssRNA. In the absence of a ligand, TLR8 forms a dimer that suffers a conformational change upon activation by recognition of its target [13]. Different from other TLRs, TLR8 forms a ring-shaped structure [12], which requires proteolytic cleavage at the Z-loop region [14]. Between species, the ectodomain (LRRs) determines whether signaling is initiated in response to a ligand stimulation, and within the ectodomain the RQSYA motif has shown to be essential for the TLR8 activation [15]. Although the sequence-specific recognition of RNA by TLR8 has not been fully established, it is known that TLR8 senses ssRNA throughout its ability to form secondary structures [16] and by recognizing ssRNA AU- and GU-rich sequences [17]. That differentiates it from TLR7, which is activated by GU-rich sequences [18]. In both cases, TLR7 and TLR8 use the same ligand-binding site, but with different amino acid composition [12]. Apparently, TLR8 is able to distinguish the host RNA by nucleoside modifications and only activates the signaling response when a non-modified RNA enters the cell [19]. TLR8 is also suggested to be a vita-PAMP receptor that is able to recognize microbial structures from viable microbes [20], poly(A)/T sequences [21] or even small antiviral molecules [12]. Overall, there are several agonists identified to specifically activate TLR8 or TLR7, and those that can activate both receptors (Table 1).

Activation of TLR8 can be triggered by multiple known ligands such as viral ssRNA [4], miRNAs [16,22,23] and some agonists included in Table 1. That activation can promote co-stimulation and MHC class II expression to induce proliferation of naïve CD4 T cells [24] and Th1 differentiation [25]. In human regulatory T cells (Treg), TLR8 agonists can mediate the reversal of the suppressive function of Treg cells through the TLR8-MyD88-IRAK4 signaling pathway [11]. Additionally, TLR8 has proven to be an important driver of T follicular helper (Tfh) cell differentiation [20]. Interestingly, experimental evidence suggests that TLR8 may also have a regulatory effect on other endosomal TLRs. In fact, TLR8 is able to inhibit both TLR7 and TLR9 in in vitro cells [26]. This observation was reproduced in *Tlr8*^−/−^ mice, where the absence of TLR8 led to higher levels of expression of TLR7 and interferon-stimulated genes (ISGs), an effect that may account for an increased antiviral immunity in the infected mice [27]. 

However, whether the inhibition of TLR7 and TLR9 by TLR8 is through direct or indirect physical contact is still unknown. Furthermore, the regulation of TLR8 by other TLRs has also been suggested. In THP1 cells, a human monocyte cell line, the addition of both TLR7 and TLR8 ligands has shown an apparent inhibition of TLR8-induced cytokine expression, suggesting that TLR7 could have a modulatory effect on TLR8 responsiveness [28]. The impact of other TLRs on TLR8 expression and activation is not yet well defined, as some contradictions exist. Studies in HEK cells suggest that TLR9 inhibits TLR7, but neither TLR7 nor TLR9 inhibit TLR8 [26,29]. However, studies in mice indicate that TLR7 may negatively regulate TLR8, where the absence of TLR7 led to an upregulation of TLR8 expression, suggesting a compensatory mechanism in the immune response [30]. Future research will define the contribution of other endosomal TLRs on TLR8 function. 

## 3. TLR8 and Viral Infections

Several reports have emerged to demonstrate the importance of TLR8 in viral infections, summarized in Table 2. The strategic localization of TLR8 in the endosomes allows for the recognition of several viruses, mainly because it recognizes ssRNAs. Viral ssRNA entering the cell would colocalize into early endosomes around 15 to 20 min after infection, where the RNA could bind to TLR8 [31]. 

Nevertheless, in the case of human immunodeficiency virus (HIV), the protein SNAPIN inhibits the colocalization of TLR8 with HIV[32]. Interestingly, studies with vaccinia virus (VV) infection in murine plasmacytoid dendritic cells (pDCs) indicate that poly(A)/T-rich DNA could also be recognized by TLR8, upregulating the expression of IFN-α and IFN-β [21]. Another example of TLR8 activation by viruses is provided by Coxsackie B virus (CBV), which induced an inflammatory response mediated mostly through TLR8 and, to a lesser extent, through TLR7 [4], similar effect is observed in Influenza A Virus (H3N2) [33]. In West Nile virus (WNV)-infected bone-marrow-derived dendritic cells from *tlr8*^−/−^ mice, the lack of TLR8 resulted in an improved antiviral response due to an increase of TLR7 expression, likely as a result of a compensatory effect [27].

**Table 1:** TLR8 and TLR7 agonists.

TLR	Name	Structure	Class	Ref.
**TLR7**	*Imiquimod*	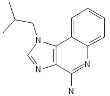	Imidazoquinoline amine analog to guanosine	[34,35]
	*Gardiquimod*	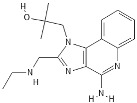	Imidazoquinoline compound	[13,36,37]
	*Loxoribine*	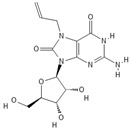	Guanosine analogue	[38,39,40,41]
	*Isatoribine*	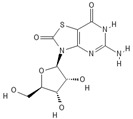	Nucleoside analogue	[42]
**TLR8**	*TL8-506*	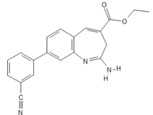	Benzoazepine compound	[43]
	*Selgantolimod*	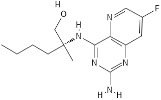	Nucleotide analogue	[44,45]
**TLR7/TLR8**	*Resiquimod*	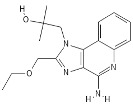	Imidazoquinoline compound derivative	[24,38,40]
	*CL075*	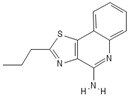	Thiazoloquinolone derivative	[29,46,47,48]
	*CL097*	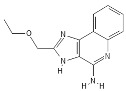	Imidazoquinoline compound derivative	[48]

TLR8 expression has been documented to increase around 2 to 6 h after some viral infections such as human parechovirus 1 (HPEV-1) [31] and influenza virus (H5N1) [49]. However, the response against viral infection after TLR8 activation highly depends on the type of cell infected. For instance, in human primary CD4+ T cells, human immunodeficiency virus 1 (HIV-1) is endocytosed by CD4 T cells and activates TLR8. This response triggers a cytokine response that helps the viral replication and reactivates latent HIV-1 through pro-inflammatory stimuli [50]. However, in human monocytes, HIV-1 exposure initially induces autophagy through a TLR8- and BECN1-dependent mechanism, an event that is eventually modulated by the productive infection of the virus [51].

**Table 2:** Effect of viruses on TLR8 response.

DNA Virus		
Virus	Effect on TLR8 Activation	Cells
**DNA Viruses**		
Vaccinia virus	Induces the expression of IFN-α, IFN-β	pDCs, HEK cells [21]
Hepatitis B virus	HBV infection inhibits innate immunity by decreasing TLR8 levels	PBMC [52]
**RNA Viruses**		
Influenza A virus (H3N2)	Induces the expression of IL-8	HEK cells, neutrophils [33]
Coxsackie B virus	Induces the expression of IFN-β, IL-6	Human cardiac cells [4]
HIV-1	Induces the expression of IL-6 and IL-1β	Human primary CD4+ T cells [50]
	Colocalization of HIV and TLR8 is decreased	Dendritic cells [32]
West Nile virus	TLR8 favors the infection in infected mice	Bone-marrow-derived dendritic cells (BMDCs) [27]
Zika virus	TLR8 and MyD88 expression decreases	Peripheral blood [53]

Studies in patients infected with viruses also suggest a critical role of TLR8 in the disease progression and immune response. In human respiratory syncytial virus (HRSV)-infected infants, low levels of TLR8 were linked to a decreased amount of TNF-α synthesis, while convalescent patients had increased to normal levels of TLR8 and increased expression of TNF-α [1]. In patients infected with Zika virus (ZV), the expression of TLR8 and MyD88 is very low in comparison to non-infected patients, which may suggest that ZV blocks, by an unknown mechanism, the expression of TLR8 [53]. A similar effect was found in patients with chronic hepatitis B virus (HBV) infection, where the expression of TLR8 in PBMCs was decreased, causing a poor cytokine and interferon expression, which could facilitate the viral replication [52]. Furthermore, mutations in TLR8 have also been reported to influence the severity of some viral diseases. Single nucleotide polymorphism (SNP) in TLR8 has shown to impair immune response and functional effects during HCV, which suggests that individuals with SNPs respond differently to viral infections [29]. Conversely, TLR8 SNPs confer a protective effect against HIV by modulating the cytokine response [54]. However, when exploring the effect of TLR8 SNP on SARS-CoV-2-infected patients with COVID-19, there was no correlation between TLR8 mutations and disease severity [55]. Overall, these correlative observations in patients might suggest that some viruses have developed an evasion mechanism that impairs the innate immune response via TLR8 inhibition and highlights the importance of TLR8 in viral infections. However, further research is warranted to define the effect of viruses on TLR8 expression and activation.

## 4. Signaling Pathways Associated with TLR8 and Its Differences with TLR7

Although both TLR7 and TLR8 recognize ssRNA, they activate different signaling pathways to promote the expression of inflammatory cytokines and interferons used in the defense against viruses (Figure 1b). Both TLR7 and TLR8 signaling pathways are mediated by the adaptor molecule MyD88, which is expressed ubiquitously in the cell cytoplasm. After a viral infection, there is a rapid redistribution of MyD88 to the endosomal compartment, where the TIR domains of TLR8 phosphorylate MyD88 [4,12]. Activation of MyD88 pathway elicits a robust inflammatory and interferon induction by the activation of TRAF6, NF-kB, IRF-7, IRF-3, AP-1, and p38 MAPK [4,25]. 

Although MyD88 has been seen as a common adaptor molecule to TLR7 and TLR8, the activation of this protein in monocytes and macrophages modulates different immune responses with different signature profiles of pro-inflammatory cytokines and type I interferon in a cell-dependent fashion [8,9,25]. In the case of pro-inflammatory cytokines, TLR7 induces more IL-1β, IL-6, IP-10 and IL-23, whereas TLR8 agonists induce more IL-1α/β, IL-6, IL-8, TNF-α, IL-12β, IL-27 and MIP-1α in myeloid dendritic cells [25,48,56]. Moreover, TLR7 agonist stimulation in monocytes fails to induce a robust NF-kB. However, it induces the activation of AP-1, which may explain the differential cytokine profile elicited by TLR8 and TLR7 agonists [25]. A similar phenomenon is observed in the production of IFN by the activation of these two receptors. While TLR7 activation induces the expression of IFN-α1 and IFN-α2 in monocytes and pDCs [56,57], TLR8 activation in monocytes and myeloid DCs expresses more IFN-β1 [8,9,25,56]. After TLR7 ligand activation, the adaptor molecule MyD88 can stimulate IRF through two different mechanisms. First, MyD88 can phosphorylate IRF7, forming a complex that promotes the IFN response. Second, MyD88 activates TRAF6, which binds to IRF7, inducing the expression of IFN-α [58]. This experimental evidence illustrates the unique characteristics of TLR8 in triggering the inflammatory and antiviral host responses.

## 5. Future Perspectives for Therapeutics with TLR8 Agonist for Viral Infections 

Commercially available TLR8 agonists have demonstrated the capability to selectively activate TLR8. There are many properties of these molecules that make them attractive candidates for use as vaccine adjuvants or antiviral activity [6,59]. These molecules can be administered through multiple methods including intravenously, oral [44], inhaled, or topically applied, which make them good candidates as therapeutical options for the future. Currently, TLR8 agonists are being investigated as vaccine adjuvants and in some other ailments such as allergy or asthma [60]. One example is that of HIV Gag protein, which was conjugated to TLR7/8 agonists as an effective way to elicit a broad-based adaptive immunity in nonhuman primates [61]. Moreover, recent work has demonstrated that the TLR7/8 agonist imidazoquinoline coupled to a novel amphiphilic carrier enhances vaccine efficiency by inducing a robust Th1 skewed antibody response in mice treated with a single shot with spike protein for SARS-CoV-2 or seasonal quadrivalent inactivated influenza virus vaccine [62]. Selgantolimod (GS-9688), also known as oral (R)-7, is a TLR8 agonist that has a good absorption properties, and is currently in phase 2 clinical trial (NCT04891770) for the treatment of chronic hepatitis B [44]. Some progress has also been done on the use of TLR8 agonists in HIV infection, where it is shown that T CD4+ TLR8 promoted Tfh differentiation towards Tfh1 and Tfh17 during HIV infection. Such differentiation and the cytokine secretion from TCD4+ via TLR8 could be exploited as a potential therapeutic target and vaccine development [50]. 

TLR8 agonists in combination with other ligands such as fms-like tyrosine kinase (Flt3L) have demonstrated to prime Ag-specific CD8+ T cells, suggesting that TLR8 ligands could also be used as potent adjuvants to prime functionally superior Ag-specific human CD8+ T cells and improve the response to viral infections [63].

## 6. Concluding Remarks

The experimental evidence reported on TLR8 highlights the relevance of this receptor in the activation of the host immunity in response to viruses. More importantly, it opens new avenues of opportunity to improve the design of current treatments for viral infections and other diseases. The use of TLR8 agonists as vaccine adjuvants, immunomodulatory therapeutics, and antiviral components represent a few excellent examples of the application of TLR8 research. However, further work is warranted to better understand the specific mechanisms that govern the expression, activation, and regulation of TLR8 by viral infections.

## Data Availability

Not applicable.
